# Reversal of endoscopic sleeve gastroplasty and conversion to sleeve gastrectomy – Two case reports

**DOI:** 10.1016/j.ijscr.2020.02.060

**Published:** 2020-02-29

**Authors:** Qiuye Cheng, Kevin Tree, Michael Edye, Michael Devadas

**Affiliations:** a Department of Surgery, Blacktown Hospital, Australia; b Discipline of Surgery, University of Western Sydney, Australia

**Keywords:** LSG, laparoscopic sleeve gastrectomy, ESG, endoscopic sleeve gastroplasty, BMI, body mass index, EWL, excess weight loss, FBC, full blood count, EUC, electrolyte/urea/creatinine, CMP, calcium/magnesium/phosphate, LFT, liver function test, ECG, electrocardiogram, CT Abdomen/pelvis, computer tomography abdomen/pelvis, TBWL, total body weight loss, Endoscopic sleeve gastroplasty, Laparoscopic sleeve gastrectomy, Weight loss surgery, Bariatric procedures

## Abstract

**Introduction:**

With the advent of more minimally invasive procedures like endoscopic sleeve gastroplasty (ESG) for weight loss and metabolic disorders, we are seeing more cases of patients presenting with sub-optimal results for consideration of alternative weight loss surgery.

The report aims to describe our experience in converting ESG to laparoscopic sleeve gastrectomy and highlight our suggested technique, challenges and pitfalls.

**Presentation of cases:**

We described two bariatrics cases detailing our findings on initial endoscopy along with methods used to reverse ESG hardware, followed by issues encountered during sleeve gastrectomy 1 month later. Case 1 being of a 33 year old female (BMI – 50.7) with previous laparoscopic band removal and 2 ESG attempts, while case 2 is a 31 year old female (BMI 44.6) with previously failed gastric balloon and ESG.

**Discussion:**

ESG reversal was performed without difficulty via endoscopy with visible sutures cut and hardware removed with snares. In both cases, the stomach was easily endoscopically distensible. During sleeve gastrectomy, extra-gastric adhesions along with more gastro-gastric sutures were encountered in case 1. In case 2, ESG hardware was noted on the external surface of stomach with misfiring of 3rd stapler reload during sleeve gastrectomy likely related to unidentified retained hardware. No post-operative complications occurred in either of the cases with adequate weight loss on one month follow up.

**Conclusion:**

In our experience, ESG conversion to sleeve gastrectomy is feasible and for the most part, uncomplicated. In our case series, we described a two staged approach to conversion although a single staged conversion is theoretically feasible.

## Highlights

• ESG conversion to sleeve gastrectomy is feasible and for the most part, uncomplicated.
• Conversion of ESG to LSG can be performed safely through a combined endoscopic-laparoscopic technique.
• Return to original stomach anatomy and a meticulous approach in removing most, if not all of the ESG hardware is required for success.

## 1 Introduction

There is currently a vast array of different modalities and treatment algorithms in managing obesity. In recent times, minimally invasive procedures such as endoscopic sleeve gastroplasty (ESG) have claimed effective weight loss without the risks of laparoscopic or open surgery [1,2]. Currently, the most popular endoscopic treatments in Australia include the intra-gastric balloon and ESG.

In Australia, the Apollo device is commonly used to perform ESG [3]. ESG is thought to induce weight loss through restricting the stomach’s ability to store food, initiate early satiety and fullness through slower gastric transit [4]. The technique uses a series of endoscopically placed transmural sutures from pre-pyloric antrum to gastro-oesophageal junction to achieve a reduction in stomach volume [4]. Current, ESG is not covered by medicare or health funds and remains self-funded in Australia.

Early studies have shown ESG to be safe with minimal post procedure complications apart from isolated case of bleeding/melaena requiring further endoscopic intervention [4,5]. Medium term results from a pioneer prospective study have shown promising results with mean excess weight loss (EWL) of 60.4% at 24 months with 85% of patients achieving the goal of >25% EWL [6]. With increasing number of ESG procedures being performed, there is also more patients who seek conversion to other bariatrics surgery like laparoscopic sleeve gastrectomy (LSG). Apart from a single twenty patient case series [7] describing technical aspects and short-term outcomes of converting ESG to LSG, long-term weight loss results or data into safety and conversion to sleeve gastrectomy is still lacking.

We present two bariatric patient whom underwent ESG conversion to LSG and describe our peri-operative/operative experiences. Our report is in line with the SCARE criteria [8].

## 2 Peri-operative work up/follow up and operative steps

Both patients underwent routine work up which included bloods (FBC, EUC, CMP, LFT, Coags, Fe, Vitamin B12/Vitamin D/folate, TFT, BSL and insulin), ECG and CT Abdomen/pelvis for identification of ESG hardware.

Conversion was performed in two stages with initial endoscopy to assess stomach pathology, determine distensibility and removal of ESG sutures/hardware. This was followed by the second stage – LSG, which involved stomach mobilization with hormonic scalpel and fashioning of gastric sleeve using a standard technique of 36 F bougie, endoGIA 60 mm stapler with imbrication of proximal staple line and omentoplexy using 3-0 prolene running and interrupted sutures respectively.

Both patients underwent routine swallow study on day two post-surgery. The discharge plans were standardised to two weeks of liquid diets and regular multi-disciplinary follow ups with the surgeon, dietician and clinical psychologist.

## 3 Case 1

A 33 years old female, BMI of 50.7 and initial weight 139.1 kg, was referred for surgical weight loss options having previously had removal of failed gastric band and sub-optimal results following two ESG attempts at a different centre. The time frame between her two ESG attempts were a year apart. Her initial BMI/weight prior to ESG was 52.7 and 144.3 kg with an excess body weight loss (EWL) of 7.1% following her ESG attempts. Her medical history included hypercholesterolemia, polycystic ovarian syndrome, insulin resistance and mild reflux.

**Fig. 1:**
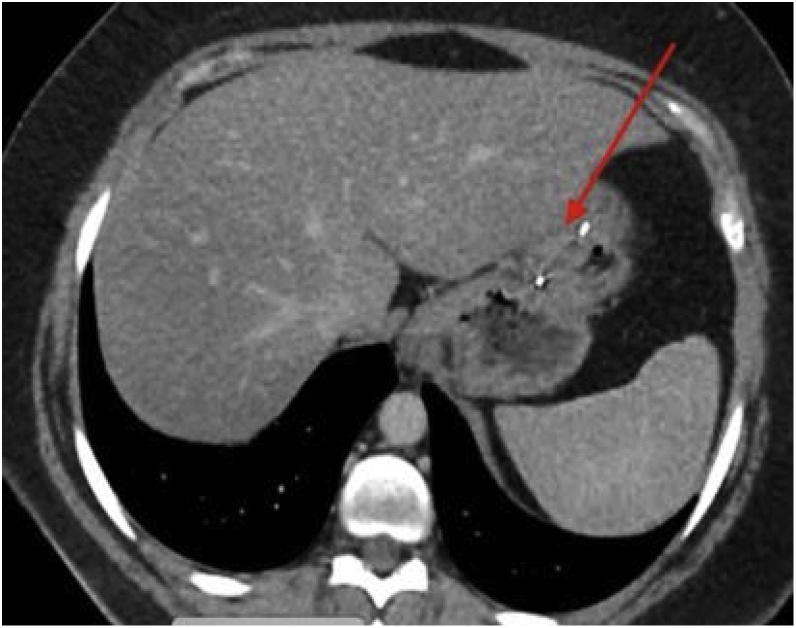
CT Axial image of deformed stomach with ESG hardware.

**Fig. 2:**
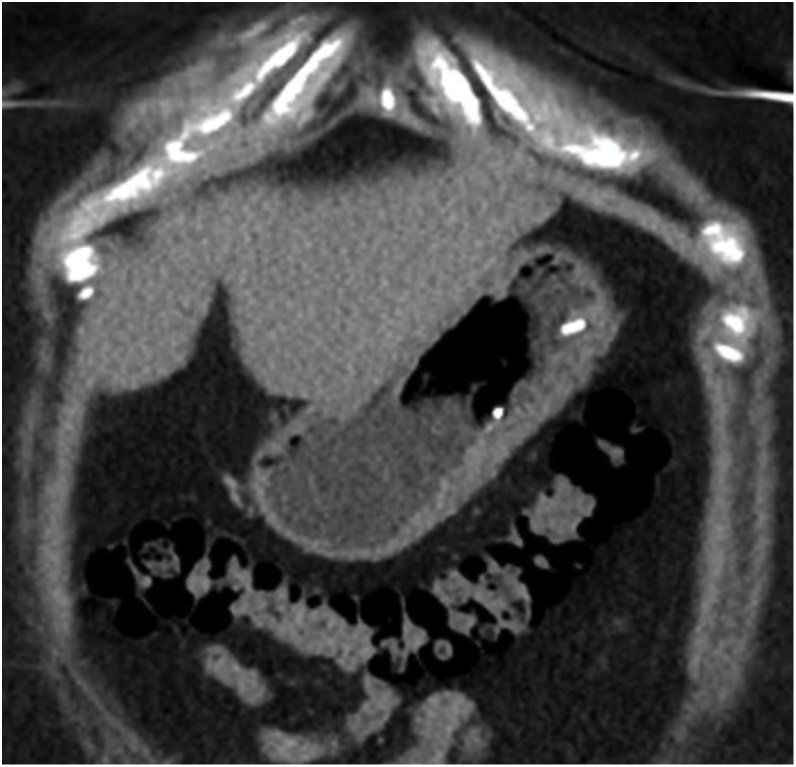
CT coronal image of ESG hardware and thickened stomach wall.

CT imaging performed demonstrated the presence of ESG hardware along with deformation and thickening of the stomach (Fig. 1, Fig. 2).

**Fig. 3:**
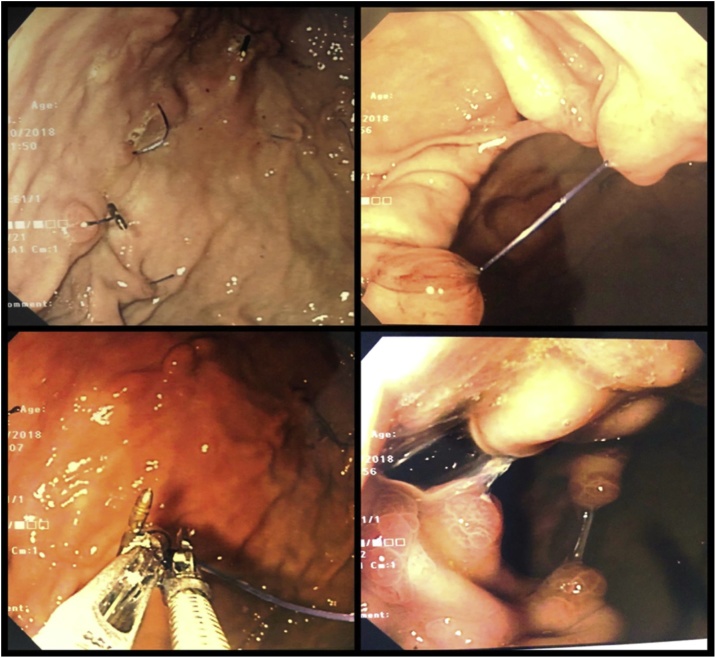
Case 1 laparoscopy showing broken ESG sutures (top left) and intact sutures (top right), removal of hardware (bottom left) and gastric inflammatory polyps (bottom right).

On initial stage endoscopy, all remaining unbroken ESG sutures were divided using endoscopic scissors. All visible ESG hardware like clips and ceramic plugs were removed with a combination of endoscopic snares and raptor graspers. A portion of the hardware was buried under gastric mucosa with evidence of neo-vascularisation and associated overlying inflammatory polyps (Fig. 3). The stomach was easily distensible prior to completion of endoscopy.

During laparoscopy, Gastro-hepatic and gastro-omental adhesions were noted and divided. A few remaining ESG sutures had to be divided to normalise stomach anatomy prior to performing LSG.

The patient had an uneventful recovery and was discharged on day three post-surgery. On Follow up, the patient has had promising weight loss results at the four months mark with BMI – 41; weight – 113.1 kg; EWL 37.0% and total body weight loss of 18.7%.

## 4 Case 2

A 31 years old female, BMI of 44.6 and initial weight of 127.5 kg, was referred for weight loss surgical options having previously had intra-gastric balloon and ESG at a different centre. Her BMI and weight prior to ESG were 47 and 134.5 kg with an EWL of 11.1%. Her medical co-morbidities included polycystic ovarian syndrome, insulin resistance and previous cholecystectomy for cholelithiasis.

On initial stage endoscopy, gastric muscular hypertrophy was noted reflecting previous gastric balloon. ESG findings were similar to case 1 with all visualised sutures, clips and cinches divided and removed as previously described.

**Fig. 4:**
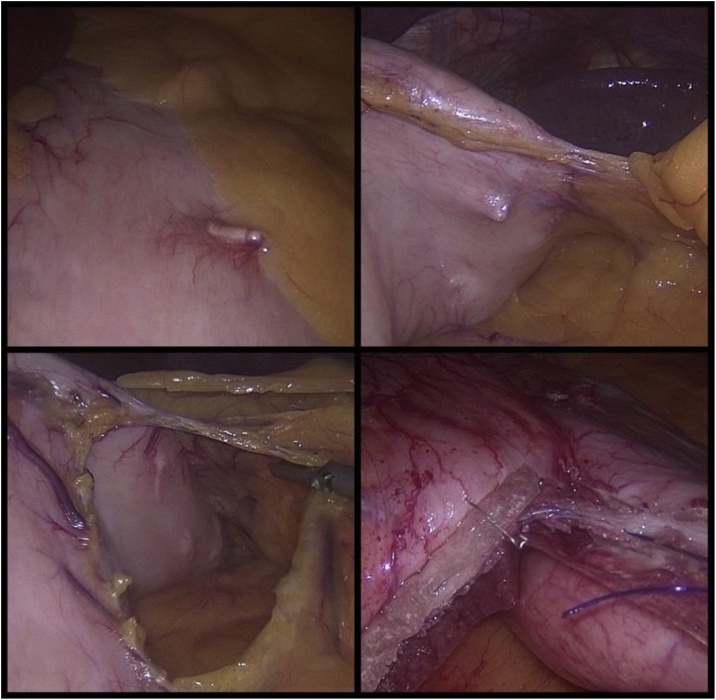
Case 2 laparoscopic findings of retained ESG hardware on external surface of stomach; buried clips and cinches and hardware in staple line (bottom right).

**Fig. 5:**
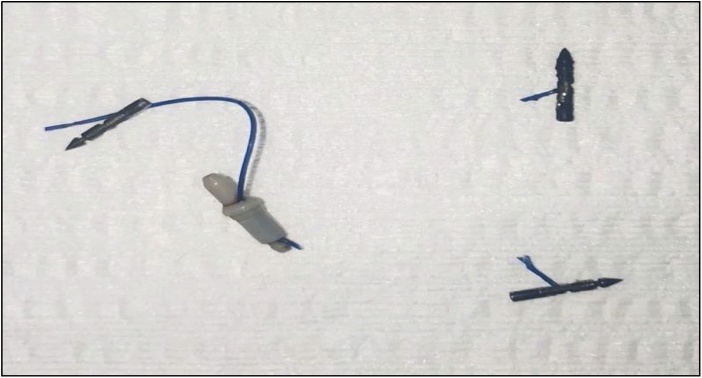
Removed ESG hardware from laparoscopy Case 2.

On initial laparoscopy, unlike the previous case, there was no significant peri-gastric adhesions or distortion of stomach anatomy. We did however note the presence of ESG hardware on the external surface of the stomach which mostly did not require removal (i.e. not in transection line) (Fig. 5). The procedure was complicated by misfiring of the third staple line requiring change of stapler secondary to previously unidentified buried ESG hardware (Fig. 4).

Like the previous case, the patient had an uncomplicated recovery and was discharged on day three. She has had good weight loss results (BMI – 36.9; weight – 105.4 kg; EWL – 39.4%; TBWL – 17.3%) at four months post-surgery.

## 5 Discussion and conclusion

The options for patients wanting to achieve weight loss can be confusing given the vast array of techniques that have developed over the last decade [9]. These two cases highlight an increasing trend for patients who initially opt for a more minimally invasive approach to weight loss but ultimately progress to laparoscopic bariatric surgery. We demonstrated that conversion of ESG is possible although challenges can arise from unexpected extra-gastric adhesions or unidentified hardware. In addition, Ferrer-Marquez et al. [10] and Movtiz et al. [11] have also described extra-gastric adhesions and traumatized/de-vascularized stomach tissue when performing reversal of ESG. In our opinion, the encountered adhesions in case 1 may have either been related to the two previous ESG attempts or prior laparoscopic gastric band insertion and subsequent removal. In this situation, adhesiolysis was uncomplicated and did not pose a challenge to revision surgery. As per our experience with case 2, the presence of undetected clips or cinches during LSG can be of significant concern as it could lead to serious complications with staple line leaks from stapler misfire if unidentified. Additionally, it appears that intra-gastric balloons can lead to a thicker stomach wall.

As a novel bariatrics procedure, ESG has gained popularity with several studies reporting on reasonable early weight loss outcomes. The two largest of these studies by Alqahtani (1000 patients with baseline BMI of 33.3 ± 4.5 kg/m^2^) and Lopez-Nava et al., 248 patients with baseline BMI of 37.8 ± 5.6 kg/m^2^) both demonstrated good short to medium-term weight loss results and reversibility of ESG if required [6,12]. However, its eventual uptake as a useful bariatrics procedure will depend on its long-term safety and weight loss results which is yet to be available. The encountered broken ESG sutures in our two cases (Fig. 3) may be of concern to the durability of sustainable weight loss through ESG which only long-term data can answer.

In our series, we have performed conversion of ESG to LSG in two stages following initial standardized work up. Moving forward, we aim to attempt conversion from ESG to LSG as a single stage endoscopic – laparoscopic operation, allowing us to compare the technical aspects/safety of either a one or two stage approach to revision. Regardless of a single or two staged approach, an endoscopic – laparoscopic approach is required. Firstly, an endoscopic evaluation is essential to assess the stomach for pathology and anatomical distortion, removal of ESG sutures and hardware (where possible) and ensure that the stomach is distensible back to its relatively normal configuration. At the conversion operation, it is important to completely mobilise stomach fundus, remove any remaining visible ESG hardware and extra-gastric adhesions to return the stomach to normal anatomy. Care needs to be taken in ensuring that the staple line trajectory is clear of hardware to avoid stapler misfiring. Another important aspect of restoring stomach anatomy is to prevent a rotated sleeve which can theoretically increase the risk of iatrogenic reflux and/or proximal staple line leak [13]. The proceduralist also needs to be aware of thickened stomach secondary to muscular hypertrophy and/or inflammatory pseudo polyps and be prepared for on table endoscopy if in doubt.

Although not used in our two cases, there may be a role for intra-operative endoscopic ultrasound (EUS) in locating undetected ESG hardware prior to LSG. Further data is required regarding EUS in order to justify its use in this situation when taking into consideration the added cost of the procedure and required technical expertise.

This case series mainly focused on ESG to LSG conversion, although we propose that the principles and challenges we encountered would apply for ESG conversions to other surgical bariatrics procedures like the roux-en Y gastric bypass or single anastomosis gastric bypass.

We will likely see an increase in number of patients with previous endoscopic treatments presenting for consideration of reversal and conversion to bariatrics surgery. Conversion of ESG to LSG can be performed safely through a combined endoscopic-laparoscopic technique. A return to original stomach anatomy and a meticulous approach to identifying and removing most, if not all of the ESG hardware is required for success.

## Sources of funding

All funding for the paper has been provided through the Department of Surgery Blacktown Hospital.

## Ethical approval

Ethics approval was obtained from the ethics committee of the Western Sydney Area Health Service.

## Consent

Written informed consent was obtained from the patient for publication of this case report and accompanying images. A copy of the written consent is available for review by the Editor-in-Chief of this journal on request.

## Author contribution

Qiuye Cheng – chief investigator and write up of manuscript.

Kevin Tree – correlation of data and preparation of figures.

Michael Edye – review and correction of manuscript.

Michael Devadas – patient information and review/correction of manuscript.

## Registration of research studies

Case report.

## Guarantor

Qiuye Cheng.

## Provenance and peer review

Not commissioned, externally peer-reviewed.

## Declaration of Competing Interest

None of the authors have any conflicts of interest to declare.
